# Teacher Satisfaction in Relationships With Students and Parents and Burnout

**DOI:** 10.3389/fpsyg.2021.703130

**Published:** 2021-09-30

**Authors:** Maria Luisa Pedditzi, Marcello Nonnis, Eraldo Francesco Nicotra

**Affiliations:** Department of Pedagogy, Psychology and Philosophy, University of Cagliari, Cagliari, Italy

**Keywords:** teacher-student relationship, educational psychology, school psychology, teacher satisfaction, teacher burnout

## Abstract

In the educational field, the role of the support component of the teacher-student relationship is well known, while the role of the teacher-student relationship on teacher burnout is a more current field of investigation. Several studies on the sources of burnout have recently focused on job satisfaction and teacher-student satisfaction. However, the role of teacher-parent satisfaction is still little explored in this field. Moreover, in the Italian school context, students’ seniority and educational level require further investigation, as the average age of teachers is particularly high compared to their European colleagues. The present study aims to examine in a sample of 882 Italian teachers the presence of burnout and differences in teacher-student and teacher-parent satisfaction between primary (students aged 6–10years) and lower secondary (students aged 11–13years) teachers. A further objective is to test whether teacher-student and teacher-parent satisfaction and seniority can be significant predictors of burnout. Teachers completed the Job Satisfaction Scale (MESI) and the MBI-Educators Survey and the data were then processed using MANOVA and multiple linear regression analysis. The results revealed that 8.2% of the teachers suffered from burnout and lower secondary teachers showed the highest levels of emotional exhaustion, depersonalisation and reduced personal accomplishment. Predictors of emotional exhaustion were job dissatisfaction and seniority, and predictors of depersonalisation were job dissatisfaction and teacher-student dissatisfaction. Finally, predictors of personal accomplishment were also teacher-parent satisfaction and teacher-student satisfaction. The implications of these findings for practice and research are discussed in this article.

## Introduction

Since the 1990s, new theoretical perspectives in education (Bruner, 1990; Lave and Wenger, 1991; Ford and Lerner, 1992; Cole, 1998) have emphasised the relational and contextualistic component of educational systems, and with the studies of Pianta (1999), the teacher-student relationship has become an independent field of investigation in educational psychology. Through various research findings in this area, it has been shown that teacher relationships can affect the quality of learning (Howes and Hamilton, 1992; Pianta, 1999; Darling-Hammond, 2006) and can alter pupils’ success- or failure-oriented trajectories (Birch and Ladd, 1996; Fraire et al., 2008; Kuriloff et al., 2019).

Several studies have also shown that the teacher-student relationship is able to influence teachers’ well-being and psychological health (Friedman, 2006; Spilt et al., 2011). The relationships that teachers establish with their students can be a source of teacher satisfaction and motivation (Hargreaves, 2000; Quan-McGimpsey et al., 2013) or a source of stress and burnout (Friedman, 2006; Corbin et al., 2019). One of the most prominent definitions describes burnout ‘as a syndrome of emotional exhaustion, depersonalization, and reduced personal accomplishment that can occur among individuals who work with people in some capacity’ (Maslach et al., 1996, p. 4). Emotional exhaustion is a feeling of tiredness and fatigue at work that leads to a feelings of reduced personal accomplishment. Depersonalisation results from attitudes of refusal to relate to the clients/patients or students and is associated with ineffective and impersonal responses to their requests (Maslach et al., 1996). Recent studies on teacher burnout consider student misbehaviour one of the main sources of the syndrome (Aloe et al., 2014) and identify the teacher-student relationship as a possible mediator between unruly student behaviour and teacher burnout (Aldrup et al., 2018). Other studies particularly focus on the conflictual nature of the teacher-student relationship as being responsible for the syndrome (Evans et al., 2019). Aldrup et al. (2018) point out that the positive quality of the teacher-student relationship is able to positively affect the increase in teachers’ well-being and work enthusiasm and protect against the potential for conflict in the teacher-student relationship (Evans et al., 2019; Klassen et al., 2012). In a recent study, Corbin et al. (2019) explored relational conflict and closeness using the teacher-student relationship scale (Pianta, 2001) in relation to burnout (Maslach et al., 1996). This study showed that relational conflict with students is able to predict teachers’ emotional exhaustion and relational closeness is able to predict personal accomplishment. Taken together, these findings are among the first to empirically support the theoretical model outlining the importance of student-teacher relationships for teacher well-being (Spilt et al., 2011).

In order to further investigate this line of research aimed at exploring the relationship between the quality of the educational relationship and burnout, this study investigated the role of teacher-student satisfaction and teacher-parent satisfaction to see if they could be considered significant predictors of the syndrome. Indeed, there are still few studies investigating this specific dimension of job satisfaction as a source of teacher burnout (Skaalvik and Skaalvik, 2009).

Job satisfaction is a pleasurable or positive emotional state resulting from the appraisal of one’s job or job experiences (Locke, 1969); it is an emotional state of well-being when there is correspondence between an individual’s characteristics (e.g., needs, expectations, and preferences) and the benefits that derive from their performances at work (Skaalvik and Skaalvik, 2010). Evans (1997) describes job satisfaction as a state of mind determined by the extent to which the individual perceives her/his job-related needs to be met. In the field of job satisfaction studies, Spector (1997) describes job satisfaction as the extent to which people like (satisfaction) or dislike (dissatisfaction) their jobs. Skaalvik and Skaalvik (2009) showed that burnout is associated with low teacher job satisfaction. Corbin et al. (2019), in a sample of German primary school teachers, highlighted the role of teacher-student relationships in predicting teachers’ personal emotional burnout. Velasco et al. (2013), in a sample of lower and upper secondary school teachers from northern Italy, also highlighted the relationship between job satisfaction and Burnout, showing a strong influence of social support on teachers’ job satisfaction and only a weak influence of managing disciplinary problems with students on burnout levels. Skaalvik and Skaalvik (2009) pointed out instead that negative relationships with students and students’ parents and lack of social support can influence teachers’ low job satisfaction and burnout onset. In considering the teacher-student and teacher-parent relationship, a variable that requires particular attention is the school level. Some studies tend in this regard to highlight the presence of higher levels of burnout among secondary school teachers working with adolescents than among teachers working at primary school level (Quattrin et al., 2009; Vercambre et al., 2009; Betoret and Artiga, 2010; Ullrich et al., 2012; Hall-Kenyon et al., 2014), while the opposite was found in other studies (Tatar and Horenczyk, 2003; Kokkinos, 2006; Tsigilis et al., 2011). Several studies conducted in Italian secondary schools tend to underline the greater conflictual nature of the relationship between teachers and pre-adolescent students, especially in the presence of unruly, turbulent, hyperactive and demotivated student behaviour in overcrowded classes (Di Pietro and Rampazzo, 1997; Pinelli et al., 1999). Some research also highlights a general discomfort in Italian secondary school teachers, which is associated with a representation of their work as predominantly individual and solitary (Buonomo et al., 2017). This would seem to be in line with their university training, which is less focused on supervision and collaboration with colleagues than primary school teachers.^1^ In addition to the school factor, another aspect that constitutes a peculiarity in Italy is the age of teachers, as the percentage of those over 50 is exceptionally higher than in other European countries. Data published by the Ministry of Education in Italy in 2016/2107 show that the average age of Italian teachers is around 51years old and the regions where the oldest teachers work are in the south where 44.2% of teachers are 54years old (OECD, 2019). Several studies have shown an age-related increase in burnout (Anastasiou and Belios, 2020; Park and Shin, 2020; Luisa et al., 2020; Polatcan et al., 2020) and in particular an increase in levels of emotional exhaustion (Pedditzi et al., 2020); other studies, in contrast, have shown that some veteran teachers can achieve fair levels of job fulfilment (Anderson, 2000; Luisa, 2015). These contradictory results made it necessary to explore seniority in order to understand the possible role of teaching experience. Here again, however, the literature shows that the data are not always consistent. Some research points to a greater vulnerability to burnout occurring when seniority of service increases due to limited energy and resources (Zavidovique et al., 2018); other research, however, notes that greater teacher experience may be associated with greater satisfaction (Veldman et al., 2013) and commitment (Ryan et al., 2017; Lowe et al., 2019). Veldman et al. (2016) also showed that in veteran teachers, the job satisfaction was positively related to the extent to which their aspirations in teacher-student relationships had been realized.

Given the not always unambiguous results concerning the above variables and their relationship with burnout, the present study aims to:

1. verify the possible presence of burnout in a sample of Italian teachers from central and southern Italy;
2. verify whether there are significant differences in burnout between primary school teachers working with children between 6 and 10years old and lower secondary school teachers working with preadolescents;
3. test whether there are significant differences in job satisfaction and teacher-student and teacher-parent satisfaction between primary and secondary teachers;
4. verify whether teacher satisfaction and in particular teacher-student and teacher-parent satisfaction and seniority of service can be significant predictors of burnout, in its components of emotional exhaustion, depersonalisation and reduced personal accomplishment.

## Materials and Methods

### Participants

882 Italian teachers participated in the research: 52.4% from primary schools (*N*=462) and 47.6% from secondary schools (*N*=420). In regard to gender and age, 84.4% of the teachers were female (*N*=744) and only 15.6% were male, all aged between 27 and 63years (mean=47.5, SD=7.98). All the teachers worked in public schools and came from central and southern Italy (18.5% from Rome; 30% from Sassari, 20.2% from Bari; and 31.3% from Cagliari). The length of service ranged from 1 to 39years (mean=19.56, SD=9.3). Participants received permission from their schools to take part in the research and completed the questionnaire individually in a paper-pencil survey during breaks at school. The sample obtained was therefore one of convenience and the response rate to the questionnaire was 75% (out of 1,200 distributed, 902 were completed, of which 882 were valid). The study was conducted according to the APA (American Psychological Association, 2002) guidelines for ethical research in psychology and the Ethics Committee of the University of Cagliari approved the research (UniCa no. 0040431, 13/02/2020 - II/9).

### Measures

The questionnaires used were: Job Satisfaction Scale (Moè et al., 2010) and Maslach Burnout Inventory-Educators Survey, MBI-ES (Maslach and Jackson, 1986) in the Italian version by Sirigatti and Stefanile (1993).

The job satisfaction scale derived from MESI – Motivations, Emotions, Strategies, Incremental beliefs of teaching (Moè et al., 2010) assesses general job satisfaction in teaching and consists of 5 items (Alpha=0.84) such as: “I am satisfied with my job” and “My working conditions are excellent.” The items are rated on a 7-point Likert scale from strongly disagree (1) to strongly agree (7). The psychometric characteristics of the Italian version of the Job Satisfaction Scale are reported in Moè et al. (2010).

In order to deepen the analysis of teachers’ satisfaction regarding specific relationships with students and parents, two more *ad hoc* items were constructed using a 7-point Likert scale (1=strongly dissatisfied; 7=fully satisfied). The items are: “I feel satisfied with my relationship with students” and “I feel satisfied with my relationship with parents” and were considered for a separate integrative evaluation with respect to the other sets of questions.

The MBI-ES (Maslach Burnout Inventory-Educators Survey by Maslach and Jackson, 1986) consists of 22 items assessable on a 6-point Likert scale and evaluates emotional exhaustion, depersonalisation, and personal accomplishment (Sirigatti and Stefanile, 1993). MBI-ES maintains its specificity for analysing teachers’ burnout. The MBI consists of 22 items and the frequency of responses was tested using a 6-point response method, where the extremes are defined by never (0) and every day (6). The scales forming the MBI are as follows:

- Emotional Exhaustion (EE), which examines the feeling of being emotionally drained and exhausted by one’s work (9 items such as: “I feel tired when I get up in the morning and have to face another day of work” and “I feel exhausted by my work”; Alpha=0.87).
- Depersonalisation (DP), which measures a cold and impersonal response towards service users (5 items such as: “I seem to treat some students as if they were objects” and “I do not really care what happens to some students”; Alpha=0.71).
- Personal Accomplishment (PA), which assesses the feeling of one’s competence and the desire to succeed at work (eight items such as: “I feel full of energy” and “I have achieved many valuable things in my work”; Alpha=0.76).

High scores on the Emotional Exhaustion (EE) and Depersonalisation (DP) scales and low scores on the Personal Achievement (PA) scale demonstrate a high degree of burnout. The psychometric characteristics of the Italian version of the MBI-ES are reported in Sirigatti and Stefanile (1993).

### Data Analysis

In the first phase of the work, reliability checks were carried out on the scales using Cronbach’s Alpha. Subsequently, to identify burnout condition, we calculated the frequency of subjects with a combination of high levels of Emotional Exhaustion, Depersonalisation, and low Personal Accomplishment scores, as suggested by the MBI-ES coding manual for Italy (Sirigatti and Stefanile, 1993). To highlight the differences in burnout related to school (primary and secondary), the MANOVA was applied on the dependent variables exhaustion, depersonalisation and personal fulfilment. Then the One-Way ANOVA was applied to find out the specific effects on the individual variables. The MANOVA was also used to test the effect of school (primary and secondary) on the teacher satisfaction variables (job satisfaction, teacher-student satisfaction, and teacher-parent satisfaction) and then the One-way ANOVA was applied with the specific variables. Pearson’s bivariate correlational analysis was then calculated to check the correlations between the variables considered (burnout scales, satisfaction scales and seniority) and in view of the regression analysis all collinearity checks were performed. Finally, multiple linear regression analysis (enter method) was carried out in order to identify whether job satisfaction, teacher-student satisfaction, teacher-parent satisfaction and seniority could be considered significant predictors of teacher emotional exhaustion. The same procedure was then applied with the same predictors^2^ to the criterion variables of depersonalisation and then personal fulfilment. The statistical significance was always set at *p*<0.01.

## Results

### Scale Reliability

The reliability of the MBI scale was calculated using Cronbach’s alpha coefficient. The data on the MBI were as follows: Emotional Exhaustion (nine items: α=0.86), Depersonalisation (five items: α=0.75), Personal Accomplishment (eight items: α=0.80). The reliability of the satisfaction scale (5 items) was α=0.76.

### Burnout Levels

Of the interviewed teachers, 29.9% (*n*=264) demonstrate a high level of emotional exhaustion; 33.8% (*n*=298) have a high level of depersonalization and 28.3% (*n*=250) show a low level of professional personal achievement. Meeting all the conditions to be diagnosed at the highest level of the syndrome (high scores simultaneously in EE and DP and low scores in PA), 8.2% (*n*=72) were found to possess burnout. Most of these teachers were female (75%), with 65.3% from lower secondary schools and 34.7% from primary schools.

### School Level and Burnout

MANOVA has shown that there was a statistically significant difference in burnout based on a teacher’s school, *F* (3, 878)=5.277, *p*<0.001; Wilk’s Λ=0.982, partial η^2^=0.018.

The one-way ANOVA conducted subsequently to assess the effect of school level (primary and lower secondary) on emotional exhaustion showed that [*F* (1, 882)=7.472; *p*<0.01] lower secondary teachers were at greater risk of emotional exhaustion (Mean=20.11, SD=12.16, *n*=420) than primary teachers (Mean=18.04, SD=10.27, *n*=462). We also found that [F (1, 882)=9.44; *p*<0.01] lower secondary teachers were more depersonalised (Mean=4.07, SD=5.07, *n*=420) than their primary colleagues (Mean=3.08, SD=4.49, *n*=462). Finally, it was found that primary school teachers (Mean=38.40, SD=7.53, *n*=462) were more accomplished [F (1, 882)=11.416; *p*<0.01] than secondary school teachers (Mean=36.60, SD=8.22, *n*=420).

### School Level and Teacher Satisfaction

MANOVA has shown that there was a statistically significant difference in teacher satisfaction based on a teacher’s school, F (3, 878)=17.511, *p*<0.0005; Wilk’s Λ=0.944, partial η^2^=0.056.

The one-way ANOVA subsequently also showed that [*F* (1, 882)=40.87; *p*<0.01] lower secondary teachers (Mean=4.85, SD=1.02, *n*=420) were less satisfied at work than their primary colleagues (Mean=5.27, SD=0.90, *n*=462). Also with regard to teacher-student satisfaction, the ANOVA showed that [*F* (1, 882)=24.14; *p*<0.01] lower secondary teachers (Mean=5.55, SD=1.24, *n*=420) were less satisfied with their students than primary teachers (Mean=5.96, SD=1.14, *n*=462). There was no significant effect of school level (primary and secondary) on teacher-parent satisfaction [*F* (1, 882)=5.32; *p*>0.01, n.s.].

### Correlations

The correlations between burnout scales, satisfaction and seniority of teachers are shown in Table 1.

**Table 1:** Pearson correlations.

		EE	DP	PA	JS	TSS	TPS	S
Emotional exhaustion – EE	Pearson	1						
	Sig.	.						
Depersonalisation – DP	Pearson	0.467^**^	1					
	Sig.	0.000	.					
Personal accomplishment – PA	Pearson	−0.342^**^	−0.436^**^	1				
	Sig.	0.000	0.000	.				
Job satisfaction – JS	Pearson	−0.358^**^	−0.387^**^	0.387^**^	1			
	Sig.	0.000	0.000	0.000	.			
Teacher-student satisfaction TSS	Pearson	−0.240^**^	−0.363^**^	0.370^**^	0.681^**^	1		
	Sig.	0.000	0.000	0.000	0.000	.		
Teacher-parent satisfaction TPS	Pearson	−0.217^**^	−0.338^**^	0.383^**^	0.670^**^	0.683^**^	1	
	Sig.	0.000	0.000	0.000	0.000	0.000	.	
Seniority of service S	Pearson	0.108^**^	0.013	0.055	−0.029	−0.056	0.035	1
	Sig.	0.001	0.702	0.102	0.391	0.094	0.300	.
Sig (2-tailed) − *N*=882								

**p<0.01.

The correlations between burnout scales (EE, DP, and RP) are significant and overall good. As expected, they are positive between emotional exhaustion and depersonalisation and negative between these scales and personal accomplishment. Correlations are moderate and negative between job satisfaction and burnout. With regard to teacher-student satisfaction, a good positive correlation is observed with job satisfaction and teacher-parent satisfaction. The correlation between teacher-student satisfaction and emotional exhaustion is negative and moderate, as is the correlation with depersonalisation. As far as teacher-parent satisfaction is concerned, there is a good positive correlation with job satisfaction and teacher-student satisfaction. Finally, with regard to seniority, a positive correlation emerges only with emotional exhaustion.

### Multiple Linear Regression Analysis

#### Predictors of Emotional Exhaustion

The following are the results of the multiple linear regression analysis (Table 2) carried out using emotional exhaustion as the criterion variable and job satisfaction, teacher-student and teacher-parent satisfaction and length of service as predictors (enter method).

**Table 2:** Regression analysis.

Scales	Beta	t	Sig
Job satisfaction	− 0.376	− 8.108	**0.0001**
Teacher-student satisfaction	0.001	−0.003	0.997 n.s.
Teacher-parent satisfaction	0.031	0.663	0.508 n.s.
Seniority of service	0.096	3.035	**0.002**
Model’s fit *p*<0.01	R=0.372	R^2^ =0.138	R^2^ adjusted=0.134
	*N* =881	F=35.158	**Sig=0.0001**

*Criteria: emotional exhaustion. Bold values: significant values for p<0.01*.

*Job satisfaction (β=−0.376; t=−8.108; Sig<0.01) and length of service (β=0.096; t=3.035; Sig<0.01) are significant predictors of emotional exhaustion (R^2^ adjusted=0.134; F=35.158; Sig=0.0001)*.

#### Predictors of Depersonalisation

The results of the multiple linear regression carried out to highlight the significant predictors of depersonalisation are shown in Table 3.

**Table 3:** Regression analysis.

Scales	Beta	t	Sig
Job satisfaction	− 0.230	− 5.069	**0.0001**
Teacher-student satisfaction	− 0.151	− 3.258	**0.001**
Teacher-parent satisfaction	−0.080	−1.751	0.080n.s.
Seniority of service	0.001	0.017	0.987n.s.
Model’s fit *p*<0.01	R=0.414	R^2^ =0.171	R^2^ adjusted=0.167
	*N* =881	F=45.244	**Sig=0.0001**

*Criteria: depersonalisation. Bold values: significant values for p<0.01*.

The predictors of depersonalization (R^2^ adjusted=0.167; *F*=45.244; Sig=0.0001) are job satisfaction (β=−0.230; *t*=− 5.069; Sig<0.01) and teacher-student satisfaction (β=−0.151; *t*=−3.258; Sig<0.01).

#### Predictors of Personal Accomplishment

The multiple linear regression analysis finally carried out to identify the predictors of teachers’ personal accomplishment among the variables of job satisfaction, teacher-student and teacher-parent satisfaction and seniority, yielded the following results presented in Table 4.

**Table 4:** Regression analysis.

Scales	Beta	*t*	Sig
Job satisfaction	0.188	4.174	**0.0001**
Teacher-student satisfaction	0.134	2.916	**0.004**
Teacher-parent satisfaction	0.164	3.610	**0.0001**
Seniority of service	0.062	2.063	0.042n.s.
Model’s fit *p*<0.01	R=0.434	R^2^ =0.188	R^2^ adjusted=0.184
	*N* =881	F=50.81	**Sig=0.0001**

*Criteria: personal accomplishment. Bold values: significant values for p<0.01*.

Job satisfaction (β=0.188; *t*=4.174; Sig<0.01), teacher-student satisfaction (β=0.134; *t*=2.916; Sig<0.01), and teacher-parent satisfaction (β=0.164; *t*=3.610; Sig<0.01) are significant predictors of teachers’ personal accomplishment (R^2^ adjusted=0.184; *F*=50.810; Sig=0.0001).

## Discussion

The results of this study revealed that 8.2% of the Italian teachers in the sample were suffering from burnout. Specifically, 29.9% had a high level of emotional exhaustion (as many as 264 teachers) and 33.8% had high depersonalisation scores (as many as 298 teachers). These results confirm once again that the professional category of teachers is at risk of burnout.

The comparison between primary and secondary school teachers shows that secondary school teachers are more at risk of burnout than primary school teachers. They were also more dissatisfied with their work and the teacher-student relationship. As already found in other research (Quattrin et al., 2009; Ullrich et al., 2012; Hall-Kenyon et al., 2014), there is evidence that working with secondary school students tires teachers more than at primary school level. Teacher-student dissatisfaction could therefore be associated with teachers’ difficulties in dealing with pre-adolescent students. However, given the complexity of the Italian school context, a multiplicity of other interacting relational and organisational factors (Buonomo et al., 2017; Pedditzi and Marcello, 2018) should also be taken into account.

In contrast, no difference was observed between primary and secondary teachers regarding satisfaction with the teacher-parent relationship. This finding, which could be further explored with qualitative research methods, highlights that the teacher-parent relationship may be an under-utilised psychosocial resource at school for promoting well-being.

Among the predictors of emotional exhaustion, multiple linear regression analysis revealed job dissatisfaction and seniority of service, confirming previous research pointing to an increase in burnout with teachers’ length of service (Zavidovique et al., 2018). These findings are also in line with previous research findings on teachers’ age (Pedditzi et al., 2020; Polatcan et al., 2020; Park and Shin, 2020; Anastasiou and Belios, 2020) and show an increase in teachers’ emotional exhaustion over time.

The predictors of depersonalisation were found instead to be dissatisfaction in the teacher-student relationship and job dissatisfaction. This result is very important because it confirms that teacher-student dissatisfaction contributes to depersonalisation. The applicative implications of this result indicate the possibility of intervening in the teacher-student relationship to improve satisfaction levels and prevent depersonalisation.

Teacher-student satisfaction has also been identified as a predictor of personal accomplishment, along with job satisfaction and teacher-parent satisfaction. From the perspective of burnout prevention, it is therefore more important than ever to promote the development of good relationships with parents and the school components that represent them and to adopt an ecological and systemic view that values all educational relationships.

This study therefore, on the one hand, confirms previous research findings on the relationship between job dissatisfaction and burnout (Skaalvik and Skaalvik, 2009; Molero Jurado et al., 2019; Robinson et al., 2019) and on the other hand, highlights original research findings regarding the predictive value of teacher-student satisfaction on depersonalization and of teacher-parent relationships on personal fulfilment.

However, it is important to consider, among the limitations of this research, the fact that teacher-student and teacher-parent satisfaction are measured through single items and therefore, in a future perspective, it is necessary to deepen these dimensions with the parallel use of Pianta’s Teacher Student Relationship Scale, through a longitudinal and experimental design, in order to also capture also possible burnout development phases. It is also necessary to remember that the results of this research are specific to the sample tested and cannot be generalised to all teachers. In fact, the use of convenience samples can lead to distortions in the selection of the group, increasing the probability that the participants are those most likely to answer the questionnaire. A further limitation of our study is that all data are self-reported and therefore not completely objective. However, this study has strengths such as the large sample size and depersonalisation data, which in our research showed acceptable values of internal consistency of the scale.

These data allowed us to analyse burnout in relation to teacher-student satisfaction, taking into account all dimensions of Maslach’s model, and not only the dimensions of emotional exhaustion and personal fulfilment as in previous research (Corbin et al., 2019).

The practical implications of this study relate to the possibility of designing teacher training and burnout prevention activities aimed at improving teacher-student and teacher-parent relationships to promote the well-being of teachers and the entire school community.

## Data Availability Statement

The raw data supporting the conclusions of this article will be made available by the authors, without undue reservation.

## Ethics Statement

The studies involving human participants were reviewed and approved by Ethics Committee of the University of Cagliari (UniCa no. 0040431, 13/02/2020 - II/9). The patients/participants provided their written informed consent to participate in this study.

## Author Contributions

MP designed and wrote the study. MN supervised the data collection. EN supervised the statistical analysis. All authors contributed to the article and approved the submitted version.

## Funding

This work was supported by the University of Cagliari (Supplementary Research Fund for 2020-FIR). The funding body has no role in the data collection of the manuscript.

## Conflict of Interest

The authors declare that the research was conducted in the absence of any commercial or financial relationships that could be construed as a potential conflict of interest.

## Publisher’s Note

All claims expressed in this article are solely those of the authors and do not necessarily represent those of their affiliated organizations, or those of the publisher, the editors and the reviewers. Any product that may be evaluated in this article, or claim that may be made by its manufacturer, is not guaranteed or endorsed by the publisher.

## Footnotes

^1^Primary school teacher training is regulated by Ministerial Decree 249/2010 and secondary school teacher training by the more recent Legislative Decree 59 of 2017.

^2^Gender was not included as a predictor because the sample is predominantly female and results on this variable vary significantly between countries in relation to gender egalitarianism (Garcia-Arroyo et al., 2019).
